# Induction of host genes by nested genes during *C. elegans* development

**DOI:** 10.1016/j.isci.2025.113021

**Published:** 2025-06-27

**Authors:** Fabien Soulavie, Carole Couillault, Matéo Melki, Khulganaa Buyannemekh, Antoine Barrière, Paul Villoutreix, Vincent Bertrand

**Affiliations:** 1 Aix Marseille University, CNRS, IBDM, Turing Center for Living Systems, Marseille, France; 2 Aix Marseille University, Université de Toulon, CNRS, LIS, Turing Centre for Living Systems, Marseille, France; 3 Aix Marseille University, INSERM, MMG, Turing Centre for Living systems, Marseille, France

**Keywords:** Genes, Developmental genetics, Developmental biology

## Abstract

Embryonic development relies on tightly controlled gene expression. In this study, we analyzed the transcriptional effect during *C. elegans* development of a striking genomic topology, the opposite nested configuration, where a gene is located in an intron of a host gene in the opposite direction. Using CRISPR genome engineering and single molecule FISH, we characterized the regulatory interactions between *ceh-10*, a transcription factor involved in neuronal specification, and its host *polq-1*, a DNA repair enzyme, showing that the nested gene induces transcription of a short version of its host in neurons. Extending our analysis to the hundreds of protein coding genes in opposite nested configuration and using single-cell RNA-seq data covering *C. elegans* embryogenesis, we observed that coexpression between nested and host genes is relatively common especially in cells positive for the nested gene. Our study illustrates how the presence of a nested gene can influence expression of its host.

## Highlights

• The host gene *polq-1* is coexpressed with its nested gene *ceh-10* in neurons
• *polq-1* transcription in neurons starts internally at the level of the *ceh-10* gene
• *polq-1* transcription in neurons is regulated by *ceh-10 cis*- and *trans-*regulators
• Coexpression of nested and host genes is relatively common during development

## Graphical abstract

**Figure:**
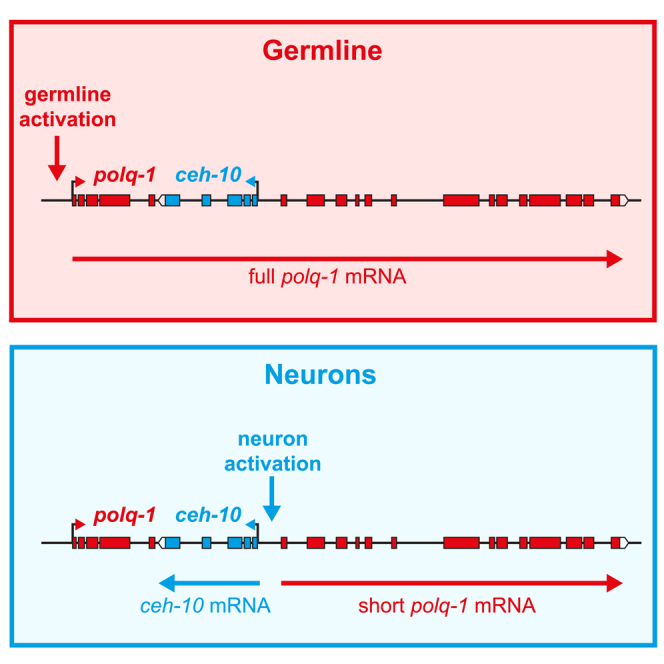

## Introduction

During animal development, gene expression is tightly regulated to support robust tissue patterning and cell fate specification.^1^^,^^2^ For key developmental genes, such as transcription factors or signaling proteins, defects in the spatial and temporal transcription pattern or in the number of mRNAs produced can lead to severe developmental failures.^1^^,^^3^^,^^4^ Specific mechanisms that ensure robustness in gene expression have been identified, such as the redundancy between *cis*-regulatory elements.^1^^,^^5^ However, the influence of many other genomic features on gene expression precision during development remains unexplored. In this study, we characterized how a particularly striking genomic configuration, the opposite nested arrangement, affects gene expression during development.

**Figure 1:**
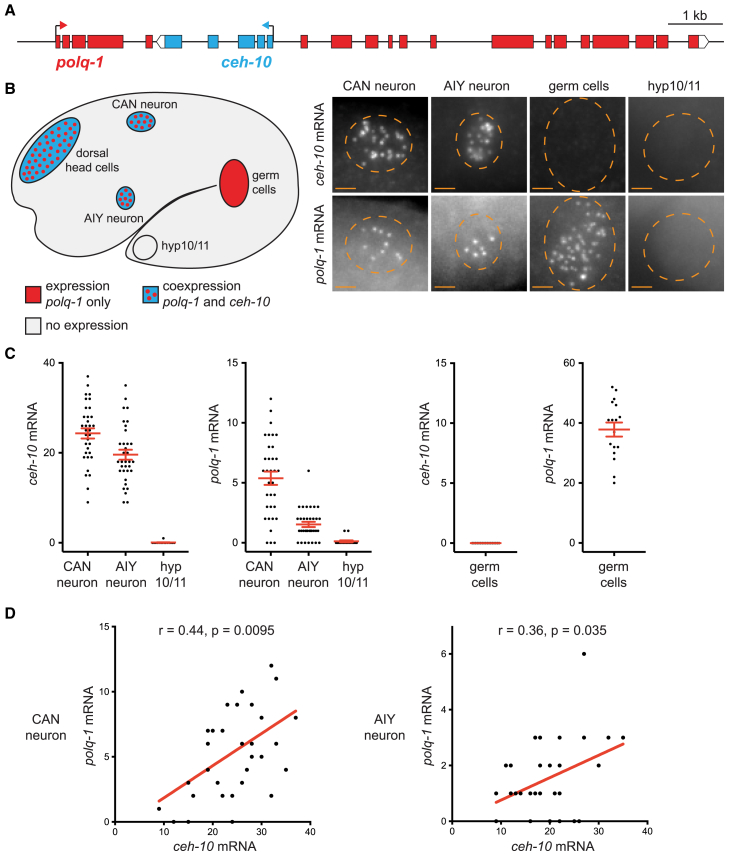
*ceh-10* and *polq-1* are coexpressed in neurons (A) Locus *polq-1/ceh-10*. (B) Scheme: expression of *ceh-10* and *polq-1* in 1.5-fold stage embryo. Pictures: co-staining of *ceh-10* and *polq-1* mRNA by smFISH with exonic probes (labeled with Quasar 670 and 570 respectively) in CAN, AIY, germ cells or hyp10/11 cells at 1.5-fold stage. The same cell is presented in *ceh-10* and *polq-1* channels. Scale bar: 2 μm. Dashed circles represent cell positions. (C) Quantification of *ceh-10* and *polq-1* mRNA numbers at 1.5-fold stage in CAN neurons, AIY neurons, hyp10/11 cells or germ cells. For CAN or AIY, each dot represents quantification in a single neuron (*n* = 34 neurons analyzed in each case). For hyp10/11 or germ cells, each dot represents quantification in a pair of neighboring cells (hyp10 + hyp11 or Z2 + Z3 respectively) (*n* = 17 pairs analyzed in each case). The red bars represent the mean and SEM. (D) Correlation between *ceh-10* and *polq-1* mRNA numbers in CAN or AIY neurons. Each dot represents a single neuron (*n* = 34 neurons analyzed in each case). *r* = Spearman’s correlation. See also Figure S1.

Protein-coding genes are in an opposite nested configuration when a gene (the nested gene) is located in an intron of another gene (the host gene) in an opposite direction (Figure 1A). The genomes of many animals, including *Caenorhabditis elegans*, *Drosophila*, and Human, contain several hundreds of protein-coding genes in an opposite nested arrangement, many coding for developmental genes.^6^^,^^7^ This configuration is a challenge for transcriptional precision as transcriptions of the host and nested genes could interfere in a positive or negative manner. For example, *in vitro* experiments have shown that transcription of the same piece of DNA in both directions at the same time can lead to collisions between polymerases transcribing in opposite directions and to a premature stop of transcription.^8^^,^^9^ In this study, we characterized how the opposite nested arrangement affects gene expression during development with single-cell resolution using *C. elegans* as an experimental system. *C. elegans* is a very good system to analyze gene expression variability and robustness during development. It has an invariant number of cells that are generated by a fixed cell lineage.^10^^,^^11^ With single molecule fluorescence *in situ* hybridization (smFISH), it is possible to quantify the number of mRNA molecules of a specific gene in each individual cell of the embryo.^12^ In addition, the recently generated comprehensive single-cell RNA-sequencing (scRNA-seq) data of *C. elegans* embryonic development now allows us to interrogate the mRNA content of every single cell at each developmental stage.^13^

Using CRISPR genome engineering and smFISH, we first dissected the interactions between the developmental gene *ceh-10* and its host *polq-1* (Figure 1A). *ceh-10* codes for a transcription factor, ortholog of VSX1 and VSX2 in vertebrates, and is involved in the specification of several neuron types, including the AIY cholinergic interneurons and the CAN osmoregulatory neurons.^14^^,^^15^^,^^16^^,^^17^ *polq-1* codes for an enzyme involved in DNA repair in the germline.^18^ Here, we show that *ceh-10* induces the expression of its host *polq-1* in neurons. Expanding the analysis to the other 566 opposite nested pairs of protein coding genes in the genome using scRNA-seq data of *C. elegans* embryonic development, we establish that coexpression between nested and host genes is not rare and suggest that nested genes are more likely to induce expression of their hosts than the converse.

## Results

### *polq-1* is transcribed in *ceh-10* positive somatic cells

To analyze whether transcriptional interactions exist between *ceh-10* and *polq-1*, we first characterized their expression by detection of their mRNAs using smFISH.^12^ *ceh-10* expression starts at mid-embryogenesis in a few neurons, epidermal cells and neuronal progenitor cells, and has been previously well characterized at the 1.5-fold stage of mid-embryogenesis.^13^^,^^14^^,^^17^^,^^19^ We therefore co-labeled 1.5-fold stage embryos by smFISH with exonic probes targeting *ceh-10* and *polq-1* mRNAs in two colors. Consistent with previous observations,^13^^,^^14^^,^^17^^,^^19^ *ceh-10* mRNAs are detected in the pair of CAN neurons, the pair of AIY neurons and a group of dorsal head cells that includes the RID neuron, the ALA neuron and the pair of epidermal cells hyp3 (Figures 1B, 1C, S1A, and S1B). As expected from the role of *polq-1* in DNA repair of the germline,^18^ *polq-1* mRNAs are present in the germ cells (Figures 1B and 1C). Surprisingly, many *polq-1* mRNAs are also detected in the soma in *ceh-10* expressing cells (CAN neurons, AIY neurons and group of dorsal head cells; Figures 1B, 1C, S1A, and S1B). On the contrary, *polq-1* mRNAs are only rarely present in *ceh-10* negative somatic cells such as the hyp10/11 epidermal cells (Figures 1B and 1C). In addition, we observed that there is significant positive correlation between the numbers of *ceh-10* and *polq-1* mRNA molecules in single CAN neurons (Figure 1D), suggesting that a high *polq-1* expression is associated with a high *ceh-10* expression. A similar positive correlation is observed in single AIY neurons (Figure 1D). Taken together these data suggest that *polq-1* expression in somatic cells may be due to its nested configuration with *ceh-10*.

To determine if coexpression of *ceh-10* and *polq-1* can also be observed at later postembryonic stages, we analyzed the recently generated single-cell RNA-seq gene expression profile of all neurons of the *C. elegans* L4 larva^20^ (Figure S1C). It was previously observed at L4/adult stages that *ceh-10* is strongly expressed in the AIY, CAN, and RID neurons with a weaker expression in the DVC neuron.^16^^,^^21^ Consistent with this, analysis of the L4 dataset reveals a high *ceh-10* expression in AIY, CAN, and RID neurons as well as weaker expression in the DVC neuron and an additional expression in the M1 neuron (Figure S1C). Interestingly, *polq-1* expression is strongly detected in AIY, CAN, and RID neurons but barely detectable in *ceh-10* negative neurons. Taken together these data show that *polq-1* and *ceh-10* are coexpressed in somatic cells at various developmental stages.

### *polq-1* and *ceh-10* are cotranscribed from the same locus

**Figure 2:**
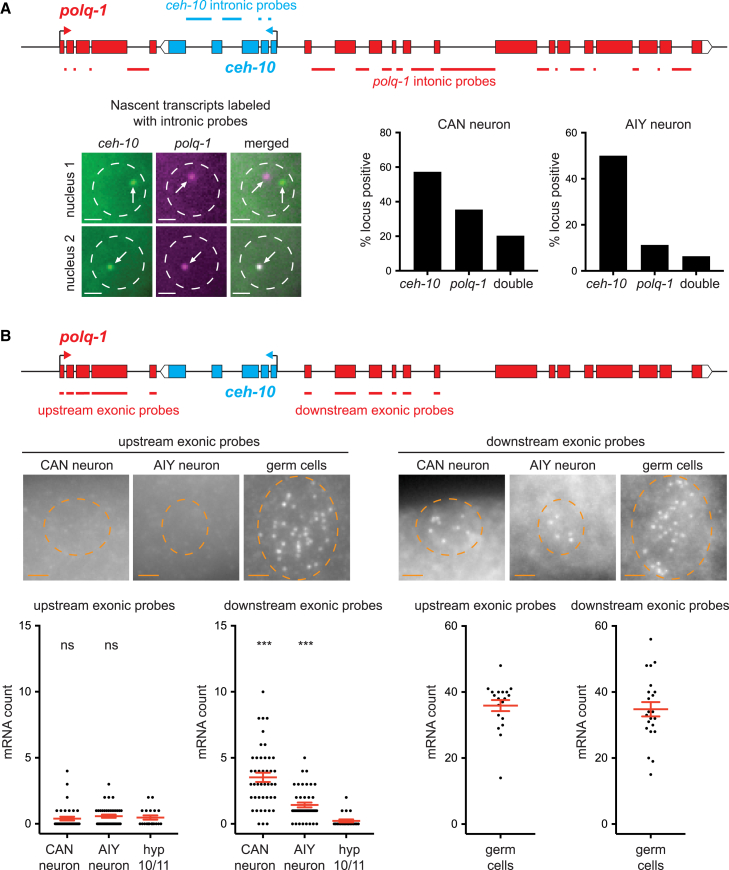
Transcription of *polq-1* in neurons starts internally (A) Detection of *ceh-10* and *polq-1* nascent RNA at transcription sites by smFISH with intronic probes (labeled with Quasar 570 and 670 respectively) at 1.5-fold embryonic stage. Pictures: CAN nucleus 1 shows one locus positive for *ceh-10* transcription and the other for *polq-1* transcription; CAN nucleus 2 shows one locus positive for both *ceh-10* and *polq-1* transcription; scale bar: 1 μm; dashed circles represent nucleus positions; arrows represent positive loci. Graph: percentage of loci positive for *ceh-10*, *polq-1* or double-positive for *ceh-10* and *polq-1* in CAN or AIY nuclei (*n* = 96 nuclei analyzed for CAN, 102 for AIY). (B) Detection of *polq-1* mRNA using exonic probes located upstream or downstream of *ceh-10* at 1.5-fold embryonic stage. Pictures: staining by smFISH in CAN, AIY or germ cells with upstream probes (labeled with Quasar 670) or downstream probes (labeled with CAL Fluor Red 610); scale bar: 2 μm; dashed circles represent cell positions. Graph: quantification of mRNA numbers (for CAN and AIY: *n* = 38 neurons analyzed for upstream, 44 for downstream; for hyp10/11 or germ cells: *n* = 19 pairs analyzed for upstream, 22 for downstream). The red bars represent the mean and SEM. Mann-Whitney U-test comparison with hyp10/11 (ns: not significant; ∗∗∗*p* < 0.005). See also Figures S2 and S3.

For CAN and AIY neurons, *ceh-10* and *polq-1* mRNAs are present in the same cells at the same time. There are two *ceh-10/polq-1* loci per cell, and thus *ceh-10* and *polq-1* could be transcribed from either distinct loci or from the same locus. To test this, we labeled in two colors *ceh-10* and *polq-1* nascent RNAs at transcription sites by smFISH with intronic probes and quantified the number of loci positive for *ceh-10*, *polq-1* or both, in CAN and AIY nuclei (Figure 2A). While some loci are positive only for *ceh-10* or *polq-1*, a notable proportion of loci is positive for both (20% in CAN, 6% in AIY), showing that *ceh-10* and *polq-1* can be transcribed from the same locus at the same time. In addition, 46% of CAN nuclei and 19% of AIY nuclei show active transcription of both *ceh-10* and *polq-1* (without considering whether intronic signal is from the same locus or not; Figure S2).

Simultaneous transcription of the same piece of DNA in both directions is problematic as it can lead to RNA polymerase II collisions.^8^^,^^9^ One possibility is that *polq-1* transcription in *ceh-10* positive somatic cells does not start at the canonical start site but more downstream, beyond *ceh-10* (Figure 2B). To test this possibility, we designed probes targeting *polq-1* exons located upstream or downstream of the intron that contains *ceh-10*. As expected, germ cells are positive for both upstream and downstream probes, indicating that the canonical full length *polq-1* mRNA is produced in germ cells (Figure 2B). However, CAN, AIY and dorsal head cells only show significant signal with downstream probes, suggesting that, in *ceh-10* positive somatic cells, a shorter version of *polq-1* mRNA is produced with a more downstream 5′ end (Figures 2B and S3).

### *polq-1* transcription in neurons is regulated by *ceh-10 trans*-regulatory factors

**Figure 3:**
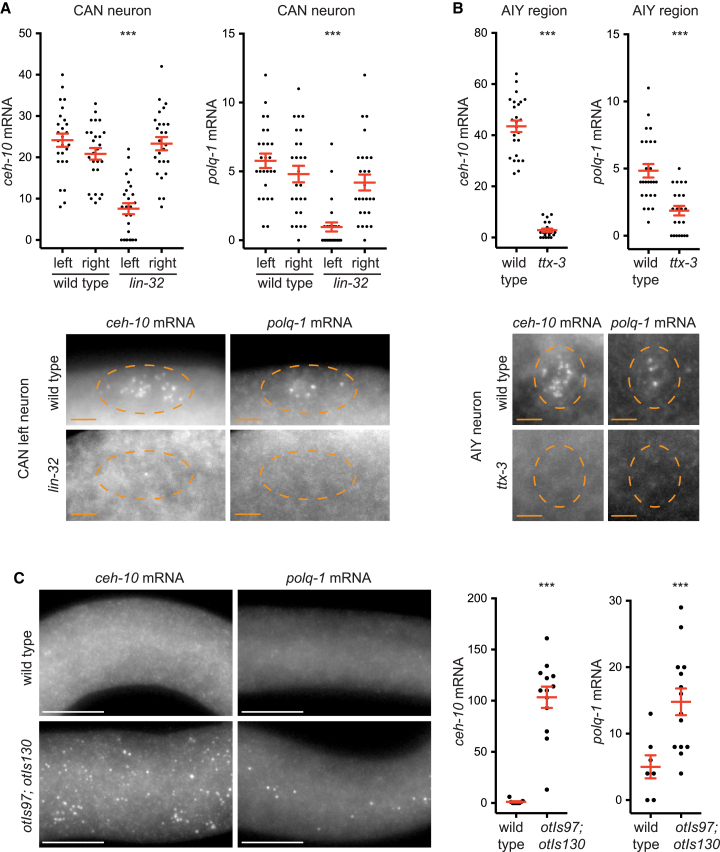
Expression of *polq-1* in neurons is regulated by *ceh-10 trans*-activators (A) Quantification of *ceh-10* and *polq-1* mRNA numbers in left and right CAN neurons at 1.5-fold embryonic stage in wild type or *lin-32(tm1446)* mutants (*n* = 26 neurons analyzed in each case, ∗∗∗*p* < 0.005 Mann-Whitney U-test). The red bars represent the mean and SEM. Pictures: co-staining of *ceh-10* and *polq-1* mRNA by smFISH with exonic probes (labeled with CAL Fluor Red 610 and Quasar 670 respectively) in the CAN left neuron at 1.5-fold stage. The same cell is presented in *ceh-10* and *polq-1* channels. Scale bar: 2 μm. Dashed circles represent cell positions. (B) Quantification of *ceh-10* and *polq-1* mRNA numbers in the AIY region at 1.5-fold embryonic stage in wild type or *ttx-3(ot22)* mutants (*n* = 25 AIY regions analyzed for wild type and 22 for mutants, ∗∗∗*p* < 0.005 Mann-Whitney U-test). The red bars represent the mean and SEM. Quantification was performed in a larger 500 μm^3^ box that contains the two AIY neurons rather than in single AIY neurons because the complete absence of *ceh-10* expression in *ttx-3(ot22)* makes the precise identification of AIY position more difficult. Pictures: co-staining of *ceh-10* and *polq-1* mRNA by smFISH with exonic probes (labeled with CAL Fluor Red 610 and Quasar 670 respectively) in the AIY neuron at 1.5-fold stage. The same cell is presented in *ceh-10* and *polq-1* channels. Scale bar: 2 μm. Dashed circles represent cell positions. (C) Detection of *ceh-10* and *polq-1* mRNA by smFISH with exonic probes at early larval stage in wild type or *otIs97[unc-119p::ttx-3]; otIs130[unc-119p::ceh-10]* transgenic animals. Pictures: region of the larva between the pharynx and the gonad; scale bar = 10 μm. Graph: quantification of mRNA numbers in a region between the pharynx and the gonad (region of 40 μm long just posterior to the pharynx) (for *ceh-10* mRNA: *n* = 9 animals analyzed for wild type and 13 for transgenic; for *polq-1* mRNA: *n* = 7 animals analyzed for wild type and 14 for transgenic; ∗∗∗*p* < 0.005 Mann-Whitney U-test). The red bars represent the mean and SEM. See also Figure S4.

We next tested whether *polq-1* transcription in somatic cells depends on upstream regulators of *ceh-10* expression. It has been previously shown that *ceh-10* expression in the left CAN neuron, but not in the right CAN neuron, is regulated by LIN-32, a proneural bHLH transcription factor of the Atonal family.^22^ Consistent with this, we observed that, in *lin-32(tm1446)* loss-of-function mutants, *ceh-10* mRNA numbers are reduced in left CAN neurons but not right CAN neurons at 1.5-fold embryonic stage (Figure 3A). Interestingly, we detected a concomitant reduction of *polq-1* mRNA numbers in left CAN neurons but not right CAN neurons (Figure 3A). We then tested whether a similar coregulation is also present in the AIY neuron, where *ceh-10* expression is regulated by the LIM-homeodomain transcription factor TTX-3.^16^^,^^17^^,^^23^ We observed that, in *ttx-3(ot22)* null mutants, both *ceh-10* and *polq-1* mRNA numbers are reduced at 1.5-fold embryonic stage (Figure 3B). We conclude that transcriptional regulators of *ceh-10* expression are also responsible for *polq-1* expression in neurons.

We then analyzed whether ectopic expression of *ceh-10* transcriptional activators would induce ectopic expression of *polq-1*. The TTX-3 and CEH-10 transcription factors cooperate to maintain *ceh-10* transcription in the AIY neuron at larval and adult stages.^15^^,^^16^^,^^17^ It was previously observed that ectopically expressing TTX-3 and CEH-10 proteins using the broadly active *unc-119* promoter leads to ectopic expression of AIY markers in the body at larval stages, especially in the epidermis.^16^ We therefore analyzed a transgenic line where *ttx-3* coding sequence and *ceh-10* cDNA are ectopically driven by the *unc-119* promoter (*otIs97[unc-119p::ttx-3]; otIs130[unc-119p::ceh-10]*). We focused on the body region located between the pharynx and the gonad where *ceh-10* mRNAs are absent in wild type animals and where the *otIs97* and *otIs130* transgenes have been reported to induce a strong ectopic expression of AIY markers.^16^ In transgenic L1 larvae, we detected ectopic *ceh-10* mRNAs in this region using exonic smFISH (Figure 3C). Using *ceh-10* intronic probes, which recognize endogenous *ceh-10* transcripts but not *ceh-10* transcripts generated by the transgene (*ceh-10* intronic sequences are absent from the transgene), we confirmed that the endogenous *ceh-10* locus is ectopically transcribed in transgenic larvae (Figure S4). Interestingly, we observed that *polq-1* expression is also ectopically induced in transgenic larvae (Figure 3C). This indicates that ectopic expression of *ceh-10 trans*-regulatory factors is sufficient to drive *polq-1* transcription.

### *polq-1* transcription in neurons is regulated by *ceh-10 cis*-regulatory regions

**Figure 4:**
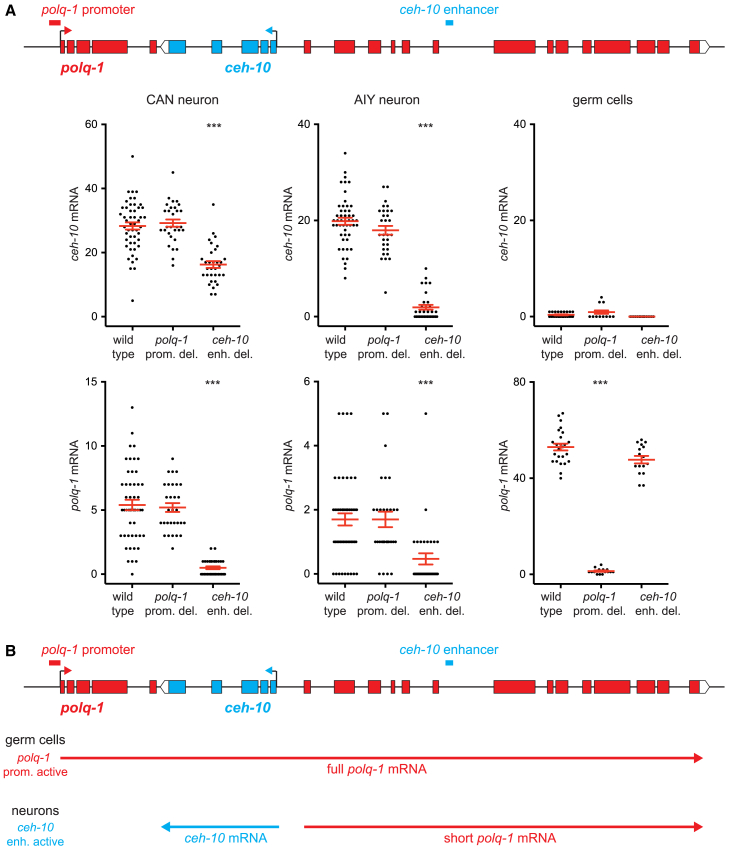
Expression of *polq-1* in neurons is regulated by a *ceh-10* enhancer (A) Quantification of *ceh-10* and *polq-1* mRNA numbers at 1.5-fold embryonic stage in wild type, *polq-1(vba29)* promoter deletion mutant or *ceh-10(vba32)* enhancer deletion mutant (for CAN and AIY: *n* = 50 neurons analyzed for wild type, 30 for *polq-1(vba29)* and 32 for *ceh-10(vba32)*; for germ cells: *n* = 25 pairs analyzed for wild type, 15 for *polq-1(vba29)* and 16 for *ceh-10(vba32)*; ∗∗∗*p* < 0.005 Mann-Whitney U-test). The red bars represent the mean and SEM. (B) Model for the activity of the *polq-1/ceh-10* locus in germ cells and neurons. See also Figures S5–S7.

We next analyzed the *cis*-regulatory regions required for *polq-1* expression in neurons. We first deleted the *polq-1* promoter region (120 bp) using CRISPR genome engineering and characterized the effect on *ceh-10* and *polq-1* expression by smFISH at 1.5-fold embryonic stage (Figure 4A). As expected, *polq-1* promoter deletion abolished *polq-1* expression in germ cells, yet it had no effect on *polq-1* or *ceh-10* expression in CAN and AIY neurons. We then deleted a *ceh-10* enhancer (54 bp) previously identified as regulating *ceh-10* expression in CAN and AIY neurons^16^^,^^17^ (Figure 4A). This enhancer contains a TTX-3 binding site. *ceh-10* enhancer deletion reduces *ceh-10* expression in CAN and AIY neurons. Interestingly, we observed a concomitant reduction of *polq-1* expression in CAN and AIY neurons, while *polq-1* expression in germ cells remains unaffected. Taken together these data suggest that, in germ cells, the canonical *polq-1* promoter is active leading to the production of full *polq-1* mRNAs (Figure 4B). In CAN and AIY neurons, the *ceh-10* enhancer is active inducing the expression of *ceh-10* mRNAs and the associated production of short *polq-1* mRNAs.

### The POLQ-1 protein has no major role in CAN and AIY neuron development

Short *polq-1* mRNAs could just be a by-product of the *ceh-10* mRNA production mechanism with no function in CAN and AIY neurons. An alternative hypothesis is that these short *polq-1* mRNAs may produce a protein with specific functions. POLQ-1 is a DNA polymerase involved in DNA repair.^18^ It contains an N-terminal helicase domain (absent from *polq-1* short mRNAs) and C-terminal polymerase domain (present in *polq-1* short mRNAs). To test whether *polq-1* short may have a function, we analyzed the effect of a deletion (*tm2026*) in the *polq-1* coding region present in *polq-1* short. This deletion has been previously reported to abolish POLQ-1 DNA repair function.^18^ As *ceh-10* regulates the formation of AIY and CAN neurons, we tested the effect of *polq-1(tm2026)* on the development of these neurons. In *ceh-10* mutants, the specification of AIY neurons is defective,^15^^,^^24^ however in *polq-1(tm2026)* mutants we did not observe defects in AIY neurons production (Figure S5A). *polq-1(tm2026)* mutants also did not enhance the loss of AIY neurons observed in *ttx-3(ks5)* weak hypomorphic mutants. In addition, *polq-1(tm2026)* mutants did not show AIY axonal defects or enhance AIY axonal defects in *ttx-3(ks5)* background. It has been previously shown that *ceh-10* regulates the specification and migration of the CAN neurons from the anterior region to the vulva region during embryogenesis.^15^^,^^25^ However, in *polq-1(tm2026)* mutants we did not observe any defects in CAN neuron production or migration (Figure S5B). This suggests that, contrary to the CEH-10 protein, the POLQ-1 protein has no major role in CAN or AIY neuron development.

### scRNA-seq data analysis reveals that coexpression of nested and host genes is not rare during *C. elegans* development

**Figure 5:**
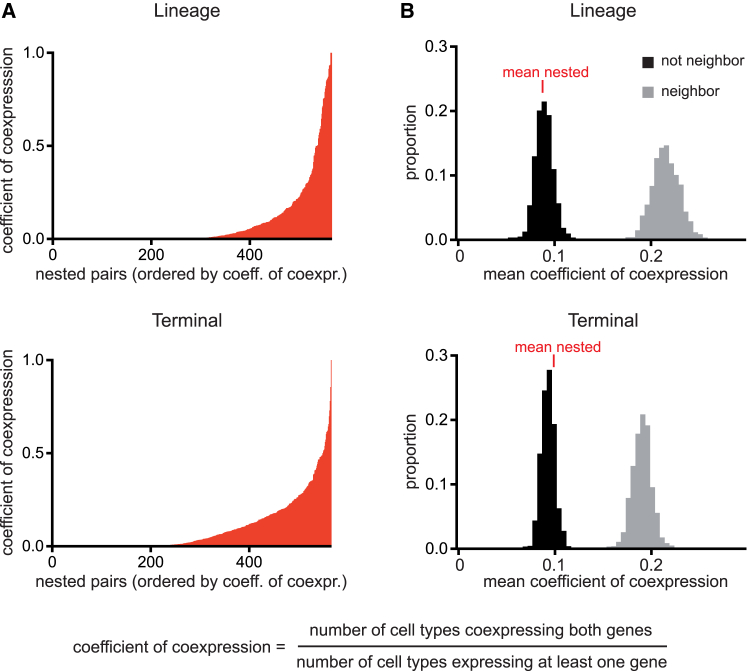
Genome-wide analysis of nested gene coexpression using scRNA-seq data (A) Coefficient of coexpression measured for each of the 567 opposite nested gene pairs. On the y axis is the coefficient of coexpression defined as the number of cell types coexpressing both genes of a pair divided by the number of cell types expressing at least one gene of the pair. On the *x* axis are the opposite nested gene pairs ordered by increasing values of the coefficient of coexpression. Lineage: complete lineage up to the terminal divisions at mid-embryogenesis; terminal: all terminal cells at different time points during later stages. (B) Histogram showing the distribution of 1000 iterated calculations of the mean coefficient of coexpression obtained from 567 pairs of random genes located on different chromosomes (not neighbor) or 567 pairs of non-nested genes located less than 1 kb apart in opposite orientation (neighbor). For comparison, the value of the mean coefficient of coexpression for the 567 opposite nested gene pairs is presented in red. See also Table S1.

Looking at the *C. elegans* genome, we identified 567 protein-coding gene pairs in an opposite nested arrangement (representing 5% of protein-coding genes in the genome). To determine whether the coexpression of nested and host genes in the same cells (as we observe with the *ceh-10*/*polq-1* pair) is a common or rare feature, we analyzed the available scRNA-seq expression profile of *C. elegans* embryonic development.^13^ These expression data cover the complete lineage up to the terminal divisions at mid-embryogenesis (lineage dataset) and all terminal cells at different time points during later stages (terminal cell dataset). To measure the degree of coexpression we calculated the coefficient of coexpression defined as the ratio between the number of cell types that coexpress both genes and the number of cell types that express at least one of the genes. We observed that more than half of nested pairs show coexpression in at least one cell type (52% for lineage dataset, 61% for terminal dataset) indicating that coexpression is not a rare feature for nested genes (Figure 5A; Table S1). We then compared the coefficient of coexpression with random pairs of genes from different chromosomes or neighboring but non-nested pairs of genes (Figure 5B). We observed that nested pairs show on average less coexpression than neighboring pairs, suggesting that coexpression usually tends to be counter-selected probably to limit transcriptional interference. However, some nested gene pairs are highly coexpressed. Finally, we noticed that the mean probability of having the host expressed in a cell type when the nested is expressed (0.50 for lineage dataset, 0.49 for terminal dataset) is higher than the mean probability of having the nested expressed when the host is expressed (0.16 for lineage dataset, 0.18 for terminal dataset) (see STAR Methods for details on calculation). This suggests that, as observed in the *ceh-10*/*polq-1* case, the nested gene is more likely to induce expression of its host than the other way around.

## Discussion

In this article, we analyzed the effect of a striking genomic configuration, the opposite nested gene arrangement, on protein coding gene expression during development. We first characterized in detail the *ceh-10*/*polq-1* gene pair. We observed that *polq-1* is not only transcribed in the germline, where it acts in DNA repair, but also in *ceh-10* positive neurons (Figure 4B). Interestingly, *polq-1* transcription in neurons does not start at the level of the *polq-1* promoter but more downstream, at the level of the *ceh-10* gene. This internal transcription is regulated by a *ceh-10* enhancer and by *ceh-10* activating transcription factors. We therefore propose that *polq-1* expression in neurons is the consequence of *ceh-10* expression. The dependence on common upstream transcription factors could explain the positive correlation that we observed between *ceh-10* and *polq-1* mRNA levels in single CAN and AIY neurons (Figure 1D). One possible origin of *polq-1* transcription in neurons is that the *ceh-10* promoter acts in a bidirectional manner. In many animals, including *C. elegans*, promoters often initiate transcription in both directions, however elongation is subsequently promoted in only one direction restricting the production of mRNAs to that direction.^26^^,^^27^^,^^28^ In the case of an opposite nested gene locus, the genomic sequence has to support both sense and antisense elongation, the presence of an internal promoter initiating transcription bidirectionally may therefore lead to productive elongation in both directions.

The production of *polq-1* mRNAs in neurons may be a simple by-product of *ceh-10* transcription with no specific function. Alternatively, *polq-1* expression could have a role in *ceh-10* positive neurons. The *polq-1* mRNA produced in neurons does not contain the helicase domain that is essential for POLQ protein family function in DNA repair.^29^^,^^30^ In addition, a small deletion in the POLQ-1 coding region, which abolishes POLQ-1 function in DNA repair,^18^ does not affect CAN or AIY neuron development. Therefore, it does not seem very likely that the POLQ-1 protein on its own has a specific function in *ceh-10* positive neurons. In addition, the presence of short *polq-1* mRNAs in neurons may not lead to the production of stable short POLQ-1 proteins. To test this possibility, we inserted an HA tag at the C-terminal end of POLQ-1 by CRISPR at the endogenous locus. The POLQ-1 protein seems expressed only at very low levels in *C. elegans* embryos, making detection difficult. Western blot analysis from embryos revealed a clear full length POLQ-1 protein but no clear shorter forms (Figure S6). However, we were not able to detect the POLQ-1 protein by immunofluorescence *in situ* in embryos probably because POLQ-1 protein expression levels are too low. Therefore, we cannot exclude that short POLQ-1 proteins may be present in neurons below our detection limit.

The short *polq-1* mRNA could have a non-coding function. In animals, long non-coding RNAs have emerged as important regulators of gene expression.^31^ The short *polq-1* mRNA could directly influence the expression of *ceh-10* in *cis*, independently of any protein coding function. For example, it has been observed that nascent non-coding RNAs can interact with transcriptional activators increasing their local concentration at regulatory sites.^32^^,^^33^ The presence of nascent short *polq-1* mRNAs could therefore promote *ceh-10* transcription by helping the local recruitment of transcriptional activators to the *ceh-10* promoter. Alternatively, there could be a competition between initiation of transcription in one direction or the other,^26^^,^^27^^,^^34^ and the transcription of short *polq-1* could therefore limit *ceh-10* expression. As with many genes in animals,^35^^,^^36^ *ceh-10* and *polq-1* transcription seems bursty in the cells where they are expressed, meaning that the two loci are not always transcriptionally active at all times. This gave us an additional opportunity to analyze the interactions between individual transcriptional events of *polq-1* and *ceh-10*. When analyzing active transcription of *ceh-10* and *polq-1* by labeling nascent transcripts with intronic probes (Figure 2A), we noticed that the frequency of double-positive loci is surprisingly close to the value expected if *ceh-10* and *polq-1* were independent (for CAN: frequency of double-positives 0.2031 compared to product of single positives 0.2029; for AIY: frequency of double-positives 0.0637 compared to product of single positives 0.0564). This suggests that, when looking at a specific time point at a single locus, the transcriptional status of one gene does not correlate in a positive or negative manner with the transcriptional status of the other gene in *cis*. In other terms, while *ceh-10* and *polq-1* are regulated by the same *trans-* and *cis*-regulators, their individual transcriptional events do not seem coordinated. This may however result from a balance between positive effects (such as promoter state favorable for RNA polymerase II recruitment) and negative effects (such as competition between initiation of transcription in one direction or the other). In absence of clear evidence for a role of short *polq-1* in neurons, we favor the hypothesis that *polq-1* expression in neurons is a by-product of *ceh-10* transcriptional regulation with no specific function. We also noticed that *polq-1* expression in neurons is weaker and more variable than in the germline, suggesting that it is more stochastic.

The nested arrangement of *polq-1* and *ceh-10* seems broadly conserved in nematodes. *ceh-10* is nested in *polq-1* in all the nematodes that we analyzed (*C. elegans*, *Caenorhabditis briggsae*, *Pristionchus pacificus*, *Onchocerca volvulus*, *Brugia malayi*, *Trichuris muris*; Figure S7). The orthologs of *polq-1* and *ceh-10* are however not nested in *Drosophila melanogaster* or humans.

Hundreds of protein coding genes are in an opposite nested configuration in the genomes of animals such as *C. elegans*, *Drosophila*, or Human.^6^ Using scRNA-seq data from *C. elegans*, we observed that coexpression of the nested gene and its host in the same cell is not rare during development. However, we noticed that opposite nested gene pairs show on average less coexpression than neighboring non-nested genes. Interestingly, previous genome-wide expression analysis at the tissue level also showed that opposite nested gene pairs tend to have more divergent expression than neighboring non-nested genes in *C. elegans*, *Drosophila*, or Human.^37^^,^^38^^,^^39^^,^^40^ Our analysis confirms this trend at the single cell level. This suggests that coexpression could be counter-selected because of transcriptional interference in the same cell between nested and host genes, for example, by collisions between polymerases transcribing in opposite directions.^8^^,^^9^ However, some nested gene pairs are highly coexpressed and we observed that the nested gene is more likely to induce the expression of its host than the converse, generalizing our conclusion on the *polq-1/ceh-10* pair. It is therefore possible that, for some other nested gene pairs, expression of the host gene in some cells could be a simple by-product of the activity of its nested gene with no specific function as suggested here for the *polq-1/ceh-10* pair.

In summary, our study adds another layer of complexity to the regulation of gene expression during development. Given that several genes involved in human diseases are in an opposite nested configuration^41^ and that spurious internal transcription in genes has been associated with cancer or aging in Human,^42^^,^^43^ our study could have potential implications for the characterization of human disorders.

### Limitations of the study

The *ceh-10* gene regulates its own expression,^15^ meaning that the CEH-10 protein activates the transcription of the *ceh-10* gene. This positive feedback loop may contribute to the activation of the *polq-1* gene in *ceh-10* positive cells. In addition, whether the short *polq-1* mRNA produced in neurons has a function and is translated in a short POLQ-1 protein is currently unclear. While it would have been interesting to fully separate the *polq-1* and the *ceh-10* genes, this is difficult to perform in a clean manner as the two genes are tightly imbricated with *ceh-10 cis*-regulatory elements and *ceh-10* coding region located in different introns of *polq-1*. Finally, the degree of coexpression varies between nested gene pairs and whether some specific genomic or structural features can explain these differences of coexpression levels remains to be investigated.

## Resource availability

### Lead contact

Requests for further information and resources should be directed to and will be fulfilled by the lead contact, Vincent Bertrand (vincent.bertrand@univ-amu.fr).

### Materials availability

All unique/stable reagents generated in this study are available from the lead contact without restriction.

### Data and code availability

• All data reported in this paper will be shared by the lead contact upon request.
• All original code has been deposited at Github at (https://github.com/VILLOUTREIXLab/nested_genes) and is publicly available as of the date of publication.
• Any additional information required to reanalyze the data reported in this paper is available from the lead contact upon request.

## Acknowledgments

We thank Andrew Saurin and members of our labs for comments on the manuscript. Some strains were provided by the *Caenorhabditis* Genetics Center (CGC), which is funded by 10.13039/100000002NIH Office of Research Infrastructure Programs (P40 OD010440). Imaging was performed on the PiCSL core facility (IBDM), member of the national infrastructure France-BioImaging supported by the 10.13039/501100001665French National Research Agency (ANR-24-INBS-0005 FBI BIOGEN). This work was funded by grants from the 10.13039/501100002915Fondation pour la Recherche Médicale (DEQ20180339160) to V.B. and the 10.13039/501100001665Agence Nationale de la Recherche (ANR-21-CE13-0007) to V.B. and P.V. The project leading to this publication has received funding from France 2030, the French Government program managed by the French National Research Agency (ANR-16-CONV-0001) and from Excellence Initiative of Aix-Marseille University - A∗MIDEX. This work was supported by the 10.13039/501100004097Fondation ARC pour la recherche sur le cancer with a fellowship (ARCDOC42023020006287) to K.B.

## Author contributions

F.S. and C.C. conducted and analyzed the experiments; M.M. and K.B. performed the bioinformatic analysis of scRNA-seq data; A.B. performed the evolutionary conservation analysis; F.S., M.M., K.B., A.B., P.V., and V.B. wrote the manuscript; P.V. and V.B. supervised the work and acquired funding.

## Declaration of interests

The authors declare no competing interests.

## STAR★Methods

### Key resources table

**Table:** 

REAGENT or RESOURCE	SOURCE	IDENTIFIER
**Antibodies**		

Anti-HA rabbit monoclonal antibody	Cell Signaling Technology	Cat#3724; RRID: AB_1549585
Anti-rabbit mouse monoclonal antibody conjugated with HRP	Santa Cruz Biotechnology	Cat#sc-2357; RRID: AB_628497
Anti-HA nanobody coupled to magnetic beads	ABclonal	Cat#AE109; RRID: AB_3698576

**Bacterial and virus strains**		

*E. coli* OP50	CGC	OP50

**Chemicals, peptides, and recombinant proteins**		

smFISH probes	BioCat	N/A
cOmplete Mini Protease Inhibitor Cocktail	Roche	Cat#11836153001
Novex Tris-Glycine Plus Midi Protein Gel, 4 to 20%	ThermoFisher Scientific	Cat#WXP42012BOX
Chemiluminescent detection reagent ECL Prime	Amersham	Cat#RPN2232

**Deposited data**		

Code	This study	https://github.com/VILLOUTREIXLab/nested_genes

**Experimental models: Organisms/strains**		

*C. elegans* N2	CGC	N2
*C. elegans* OH99: *mgIs18 IV*	Hobert Lab	OH99
*C. elegans* OH9680: *otIs130; otIs97 mgIs18 IV*	Hobert Lab	OH9680
*C. elegans* VBS708: *ceh-10(vba32) III*	This study	VBS708
*C. elegans* VBS725: *polq-1(vba33) III*	This study	VBS725
*C. elegans* VBS666: *polq-1(vba29)*/*+III*	This study	VBS666
*C. elegans* VBS667: *polq-1(vba29)*/*rhIs4 III*	This study	VBS667
*C. elegans* VBS559: *ttx-3(ot22) X*	Bertrand Lab	VBS559
*C. elegans* VBS671: *lin-32(tm1446) X*	Bertrand Lab	VBS671
*C. elegans* VBS702: *polq-1(tm2026) III; mgIs18 IV*	This study	VBS702
*C. elegans* VBS703: *mgIs18 IV; ttx-3(ks5) X*	This study	VBS703
*C. elegans* VBS704: *polq-1(tm2026) III; mgIs18 IV; ttx-3(ks5) X*	This study	VBS704
*C. elegans* VBS724: *kyIs5*	Bertrand Lab	VBS724
*C. elegans* VBS709: *polq-1(tm2026) III; kyIs5*	This study	VBS709

**Software and algorithms**		

Nikon Imaging Software	Nikon	https://www.microscope.healthcare.nikon.com/products/software/nis-elements
MATLAB	MathWorks	https://www.mathworks.com/products/matlab.html
Fiji	N/A	https://imagej.net/software/fiji/
Python	N/A	https://www.python.org
Prism	GraphPad	https://www.graphpad.com/features

### Experimental model and study participant details

Experiments were performed on *C. elegans* hermaphrodites maintained under standard laboratory conditions.^44^ *C. elegans* was grown on NGM plates and fed with *E. Coli* OP50 bacteria. The stage of *C. elegans* analyzed is presented in the figure legends. The strains of *C. elegans* used in this study are presented in the key resources table. All analyses were conducted on hermaphrodites.

### Method details

#### Genetics

The *polq-1(vba29)* promoter deletion strain, the *ceh-10(vba32)* enhancer deletion strain and the *polq-1(vba33)* HA-tagged strain were generated by CRISPR genome engineering using a *rol-6* co-conversion method.^45^ For *vba33*, a SGGGGS linker followed by 3 HA tags were inserted at the C-terminal end of POLQ-1. The deleted or inserted sequences and the sequences targeted by the guide RNAs are presented in Table S2.

#### smFISH

Stellaris smFISH probes coupled with Quasar 670, Quasar 570 or CAL Fluor Red 610 were ordered from BioCat. The probe sequences are presented in Table S3. smFISH staining on embryos and larvae was performed on slides following a previously developed protocol.^19^ Images were acquired on a Nikon Ti2E microscope equipped with a 100× 1.45 NA objective and an Andor Ikon-M high sensitivity camera using NIS (Nikon Imaging Software). Quantification of smFISH experiments using widefield microscopy is standard in the *C. elegans* field.^46^ mRNA numbers were quantified using a previously developed MATLAB script.^47^ Cells were identified by their stereotyped position.

The effect of ectopic activation of the AIY differentiation program on *ceh-10* and *polq-1* expression was analyzed with the transgenic line (*otIs97[unc-119p::ttx-3]; otIs130[unc-119p::ceh-10]; mgIs18[ttx-3p::gfp]*). As ectopic activation of the AIY differentiation program is only observed in a fraction of the animals, larvae with strong ectopic activation of the AIY differentiation program were first selected by smFISH with probes targeting *gfp* coupled with Quasar 570. On these selected larvae, *ceh-10* or *polq-1* expression was then analyzed with probes coupled with Quasar 670.

#### Imaging

Standard observations were performed under a Nikon Ti2E microscope equipped with a Hamamatsu ORCA-Flash4.0 camera and NIS (Nikon Imaging Software). Images were analyzed using Fiji.

#### Immunoprecipitation and western blot

For each strain, 10 NGM plates (9 cm in diameter) full of gravid worms were used per experiment. Embryos were extracted by treatment with a bleach solution (0.5 M NaOH, 0.8% sodium hypochlorite), then washed twice in M9 and twice in cold 4°C water. The embryo pellet was resuspended in cold 4°C lysis buffer (0.5% Nonidet P40 substitute, 50 mM Tris/HCl pH7.4, 100 mM KCl, 1 mM MgCl2, 1 mM EGTA, 10% Glycerol, 1 mM DTT, cOmplete Mini Protease Inhibitor Cocktail (Roche #11836153001)), and stored at -80°C. To extract proteins, the sample was thawed on ice and sonicated with a Diagenode Bioruptor (an average of 15 cycles, 30 sec ON - 30 sec OFF, amplitude HIGH, 4°C). The lysate was then centrifuged at 4°C at maximum speed to remove debris, and protein concentration was quantified. Before western blot, HA-tagged proteins were enriched by immunoprecipitation. Immunoprecipitation was performed starting with 3 mg of total proteins in 500 μL of lysis buffer. First, 500 μL of law salt buffer (50 mM NaCl, 150 mM Tris/HCl pH 7.4, 0.05% Tween20) was added to the sample. The sample was then incubated overnight under agitation at 4°C with 30 μL of anti-HA nanobody coupled to magnetic beads (ABclonal #AE109). After centrifugation and 2 steps of washing in law salt buffer, immunoprecipitated proteins were eluted with 40 μL of SDS buffer (1% SDS in TE, 150 mM NaCl). Leammli buffer was added and the sample boiled. Proteins were separated on a precast Novex Tris-Glycine Plus Midi Protein Gel, 4 to 20% (ThermoFisher Scientific # WXP42012BOX) and transferred to a nylon membrane. HA-tagged proteins were revealed using a primary anti-HA rabbit monoclonal antibody (Cell Signaling Technology #3724) at 1/500 and a secondary anti-rabbit mouse monoclonal antibody conjugated with HRP (Santa Cruz Biotechnology #sc-2357) at 1/1000. The HRP signal was detected using the chemiluminescent detection reagent ECL Prime (Amersham #RPN2232).

#### Bioinformatic analysis of nested genes

To identify the opposite nested gene pairs in the *C. elegans* genome, we used the Wormbase annotation version WS290. We only considered protein coding genes. For each gene in the genome, we checked if there is another gene entirely included within its genomic coordinates and in opposite orientation. If there are several nested genes within a given host, we considered each host/nested pair separately.

The scRNA-seq data were obtained from a previous study.^13^ Two datasets were used. The terminal cell dataset contains the average expression level of each gene in each annotated terminal cell type, for several different windows of embryo time. The lineage dataset contains the average expression level of each gene in each lineage annotation. The adjusted tpm (transcripts per million) value was used as a measure of gene expression. For each nested gene pair, we calculated the coefficient of coexpression. The coefficient of coexpression was defined as the ratio between the number of cell types that coexpress both genes and the number of cell types that express at least one of the genes. A gene is considered expressed if its adjusted tpm value is higher than 0. If the number of cell types that express at least one of the gene is 0 then the coefficient of coexpression is set to 0. We also estimated the coefficient of coexpression for pairs of genes located on different chromosomes, and for pairs of neighboring but non-nested genes. Pairs of genes located on different chromosomes were obtained by drawing a random gene and then associating it with another random gene from a different chromosome. Pairs of neighboring non-nested genes were obtained by drawing a random gene and associating it with another gene less than 1 kb away and in opposite orientation. The mean coefficient of coexpression was calculated from 567 pairs and the process was iterated 1000 times to generate a histogram showing the distribution of the 1000 mean coefficients of coexpression obtained. Iterations were performed to provide a representative sampling of the distribution of mean values across samples of neighboring and non-neighboring genes of size identical to the nested genes (567 pairs). Finally, for each pair, the probability of having the host gene expressed in a cell type when the nested gene is expressed was calculated by dividing the number of cell types coexpressing host and nested genes by the number of cell types expressing the nested gene. Conversely, the probability of having the nested gene expressed in a cell type when the host gene is expressed was calculated by dividing the number of cell types coexpressing host and nested genes by the number of cell types expressing the host gene.

The scripts developed for the analysis were written in Python 3.10.13 and are available as a Jupyter notebook. They use the classical scientific computing libraries pandas, numpy, scipy and plotly. They are accessible on the following Github repository: https://github.com/VILLOUTREIXLab/nested_genes.

#### Evolutionary conservation analysis

To investigate conservation of *polq-1/ceh-10* nested arrangement across nematode species, we used the following genome assemblies: *C. briggsae* (PRJNA10731), *P. pacificus* (PRJNA12644), *O. volvulus* (PRJEB513), *B. malayi* (PRJNA10729) and *T. muris* (PRJEB126). Based on gene annotations, the ortholog of *ceh-10* in *C. briggsae*, *P. pacificus* and *O. volvulus* is nested in the same position of the *polq-1* ortholog in those species. For *B. malayi* and *T. muris*, while the *ceh-10* ortholog was annotated as a single gene, *polq-1* appeared to be annotated as two paralogs (Bma16901 and Bma6150, and Tmue_2000006799 and Tmue_2000006800, respectively). However, Bma16901 and Tmue_2000006799 match the 5’ part of *polq-1*, and Bma6150 and Tmue_000002000006800 match the 3’ part. In both cases the *ceh-10* ortholog is located in inverse orientation between these two gene predictions. These split sequences therefore likely correspond to the same gene, *Bma-polq-1* and *Tmu-polq-1* respectively.

### Quantification and statistical analysis

Statistical analyses were performed using GraphPad Prism. For comparisons of mRNA numbers, a non-parametric two-tailed Mann-Whitney U-test was used: not significant (ns) p>0.5, ∗ p<0.5, ∗∗ p<0.01, ∗∗∗ p<0.005. Correlation analyses were performed using Spearman’s correlation. The numbers of animals or cells analyzed as well as the test used, p-value obtained and type of error bars are presented in the figures and figure legends.

## Supplemental information

Document S1. Figures S1–S7 and Tables S2 and S3

Table S1. Excel file, list of opposite nested gene pairs with coefficient of coexpression, related to Figure 5 (A) For lineage data. (B) For terminal data.

## References

[1] Lagha M., Bothma J.P., Levine M. (2012). Mechanisms of transcriptional precision in animal development. Trends Genet..

[2] Bentovim L., Harden T.T., DePace A.H. (2017). Transcriptional precision and accuracy in development: from measurements to models and mechanisms. Development.

[3] van der Lee R., Correard S., Wasserman W.W. (2020). Deregulated regulators: disease-causing cis variants in transcription factor genes. Trends Genet..

[4] Naqvi S., Kim S., Hoskens H., Matthews H.S., Spritz R.A., Klein O.D., Hallgrímsson B., Swigut T., Claes P., Pritchard J.K., Wysocka J. (2023). Precise modulation of transcription factor levels identifies features underlying dosage sensitivity. Nat. Genet..

[5] Eling N., Morgan M.D., Marioni J.C. (2019). Challenges in measuring and understanding biological noise. Nat. Rev. Genet..

[6] Kumar A. (2009). An overview of nested genes in eukaryotic genomes. Eukaryot. Cell.

[7] Wright B.W., Molloy M.P., Jaschke P.R. (2022). Overlapping genes in natural and engineered genomes. Nat. Rev. Genet..

[8] Crampton N., Bonass W.A., Kirkham J., Rivetti C., Thomson N.H. (2006). Collision events between RNA polymerases in convergent transcription studied by atomic force microscopy. Nucleic Acids Res..

[9] Hobson D.J., Wei W., Steinmetz L.M., Svejstrup J.Q. (2012). RNA polymerase II collision interrupts convergent transcription. Mol. Cell.

[10] Sulston J.E., Horvitz H.R. (1977). Post-embryonic cell lineages of the nematode, Caenorhabditis elegans. Dev. Biol..

[11] Sulston J.E., Schierenberg E., White J.G., Thomson J.N. (1983). The embryonic cell lineage of the nematode Caenorhabditis elegans. Dev. Biol..

[12] Raj A., van den Bogaard P., Rifkin S.A., van Oudenaarden A., Tyagi S. (2008). Imaging individual mRNA molecules using multiple singly labeled probes. Nat. Methods.

[13] Packer J.S., Zhu Q., Huynh C., Sivaramakrishnan P., Preston E., Dueck H., Stefanik D., Tan K., Trapnell C., Kim J. (2019). A lineage-resolved molecular atlas of C. elegans embryogenesis at single-cell resolution. Science.

[14] Svendsen P.C., McGhee J.D. (1995). The C. elegans neuronally expressed homeobox gene ceh-10 is closely related to genes expressed in the vertebrate eye. Development.

[15] Forrester W.C., Perens E., Zallen J.A., Garriga G. (1998). Identification of Caenorhabditis elegans genes required for neuronal differentiation and migration. Genetics.

[16] Wenick A.S., Hobert O. (2004). Genomic cis-regulatory architecture and trans-acting regulators of a single interneuron-specific gene battery in C. elegans. Dev. Cell.

[17] Bertrand V., Hobert O. (2009). Linking asymmetric cell division to the terminal differentiation program of postmitotic neurons in C. elegans. Dev. Cell.

[18] Muzzini D.M., Plevani P., Boulton S.J., Cassata G., Marini F. (2008). Caenorhabditis elegans POLQ-1 and HEL-308 function in two distinct DNA interstrand cross-link repair pathways. DNA Repair.

[19] Bordet G., Couillault C., Soulavie F., Filippopoulou K., Bertrand V. (2022). PRC1 chromatin factors strengthen the consistency of neuronal cell fate specification and maintenance in C. elegans. PLoS Genet..

[20] Taylor S.R., Santpere G., Weinreb A., Barrett A., Reilly M.B., Xu C., Varol E., Oikonomou P., Glenwinkel L., McWhirter R. (2021). Molecular topography of an entire nervous system. Cell.

[21] Reilly M.B., Tekieli T., Cros C., Aguilar G.R., Lao J., Toker I.A., Vidal B., Leyva-Díaz E., Bhattacharya A., Cook S.J. (2022). Widespread employment of conserved C. elegans homeobox genes in neuronal identity specification. PLoS Genet..

[22] Masoudi N., Yemini E., Schnabel R., Hobert O. (2021). Piecemeal regulation of convergent neuronal lineages by bHLH transcription factors in C. elegans. Development.

[23] Filippopoulou K., Couillault C., Bertrand V. (2021). Multiple neural bHLHs ensure the precision of a neuronal specification event in C. elegans. Biol. Open.

[24] Altun-Gultekin Z., Andachi Y., Tsalik E.L., Pilgrim D., Kohara Y., Hobert O. (2001). A regulatory cascade of three homeobox genes, ceh-10, ttx-3 and ceh-23, controls cell fate specification of a defined interneuron class in C. elegans. Development.

[25] Forrester W.C., Garriga G. (1997). Genes necessary for C. elegans cell and growth cone migrations. Development.

[26] Wei W., Pelechano V., Järvelin A.I., Steinmetz L.M. (2011). Functional consequences of bidirectional promoters. Trends Genet..

[27] Grzechnik P., Tan-Wong S.M., Proudfoot N.J. (2014). Terminate and make a loop: regulation of transcriptional directionality. Trends Biochem. Sci..

[28] Janes J., Dong Y., Schoof M., Serizay J., Appert A., Cerrato C., Woodbury C., Chen R., Gemma C., Huang N. (2018). Chromatin accessibility dynamics across C. elegans development and ageing. eLife.

[29] Mateos-Gomez P.A., Kent T., Deng S.K., McDevitt S., Kashkina E., Hoang T.M., Pomerantz R.T., Sfeir A. (2017). The helicase domain of Poltheta counteracts RPA to promote alt-NHEJ. Nat. Struct. Mol. Biol..

[30] Ramsden D.A., Carvajal-Garcia J., Gupta G.P. (2022). Mechanism, cellular functions and cancer roles of polymerase-theta-mediated DNA end joining. Nat. Rev. Mol. Cell Biol..

[31] Ferrer J., Dimitrova N. (2024). Transcription regulation by long non-coding RNAs: mechanisms and disease relevance. Nat. Rev. Mol. Cell Biol..

[32] Sigova A.A., Abraham B.J., Ji X., Molinie B., Hannett N.M., Guo Y.E., Jangi M., Giallourakis C.C., Sharp P.A., Young R.A. (2015). Transcription factor trapping by RNA in gene regulatory elements. Science.

[33] Oksuz O., Henninger J.E., Warneford-Thomson R., Zheng M.M., Erb H., Vancura A., Overholt K.J., Hawken S.W., Banani S.F., Lauman R. (2023). Transcription factors interact with RNA to regulate genes. Mol. Cell.

[34] Shearwin K.E., Callen B.P., Egan J.B. (2005). Transcriptional interference--a crash course. Trends Genet..

[35] Leyes Porello E.A., Trudeau R.T., Lim B. (2023). Transcriptional bursting: stochasticity in deterministic development. Development.

[36] Lee C., Shin H., Kimble J. (2019). Dynamics of Notch-Dependent Transcriptional Bursting in Its Native Context. Dev. Cell.

[37] Chen N., Stein L.D. (2006). Conservation and functional significance of gene topology in the genome of Caenorhabditis elegans. Genome Res..

[38] Ho M.R., Tsai K.W., Lin W.C. (2012). A unified framework of overlapping genes: towards the origination and endogenic regulation. Genomics.

[39] Lee Y.C.G., Chang H.H. (2013). The evolution and functional significance of nested gene structures in Drosophila melanogaster. Genome Biol. Evol..

[40] Assis R. (2016). Transcriptional interference promotes rapid expression divergence of drosophila nested genes. Genome Biol. Evol..

[41] Yu P., Ma D., Xu M. (2005). Nested genes in the human genome. Genomics.

[42] Sen P., Donahue G., Li C., Egervari G., Yang N., Lan Y., Robertson N., Shah P.P., Kerkhoven E., Schultz D.C. (2023). Spurious intragenic transcription is a feature of mammalian cellular senescence and tissue aging. Nat. Aging.

[43] Zhao Z., Chen Y., Cheng X., Huang L., Wen H., Xu Q., Zhou X., Zhang X., Chen J., Ni T. (2023). The landscape of cryptic antisense transcription in human cancers reveals an oncogenic noncoding RNA in lung cancer. Sci. Adv..

[44] Stiernagle T. (2006).

[45] Arribere J.A., Bell R.T., Fu B.X.H., Artiles K.L., Hartman P.S., Fire A.Z. (2014). Efficient marker-free recovery of custom genetic modifications with CRISPR/Cas9 in Caenorhabditis elegans. Genetics.

[46] Ji N., van Oudenaarden A. (2012).

[47] Katsanos D., Koneru S.L., Mestek Boukhibar L., Gritti N., Ghose R., Appleford P.J., Doitsidou M., Woollard A., van Zon J.S., Poole R.J., Barkoulas M. (2017). Stochastic loss and gain of symmetric divisions in the C. elegans epidermis perturbs robustness of stem cell number. PLoS Biol..

