# Supplementary material for: Induction of host genes by nested genes during C. elegans development
Source: iScience. 2025 Jun 27;28(8):113021. doi: 10.1016/j.isci.2025.113021 (PMC12274878; doi:10.1016/j.isci.2025.113021)
Supplement: Document S1. Figures S1–S7 and Tables S2 and S3 [file mmc1.pdf]

iScience, Volume *28*

Supplemental information

Induction of host genes by nested genes during *C. elegans* development

Fabien Soulavie, Carole Couillault, Matéo Melki, Khulganaa Buyannemekh, Antoine Barrière, Paul Villoutreix, and Vincent Bertrand

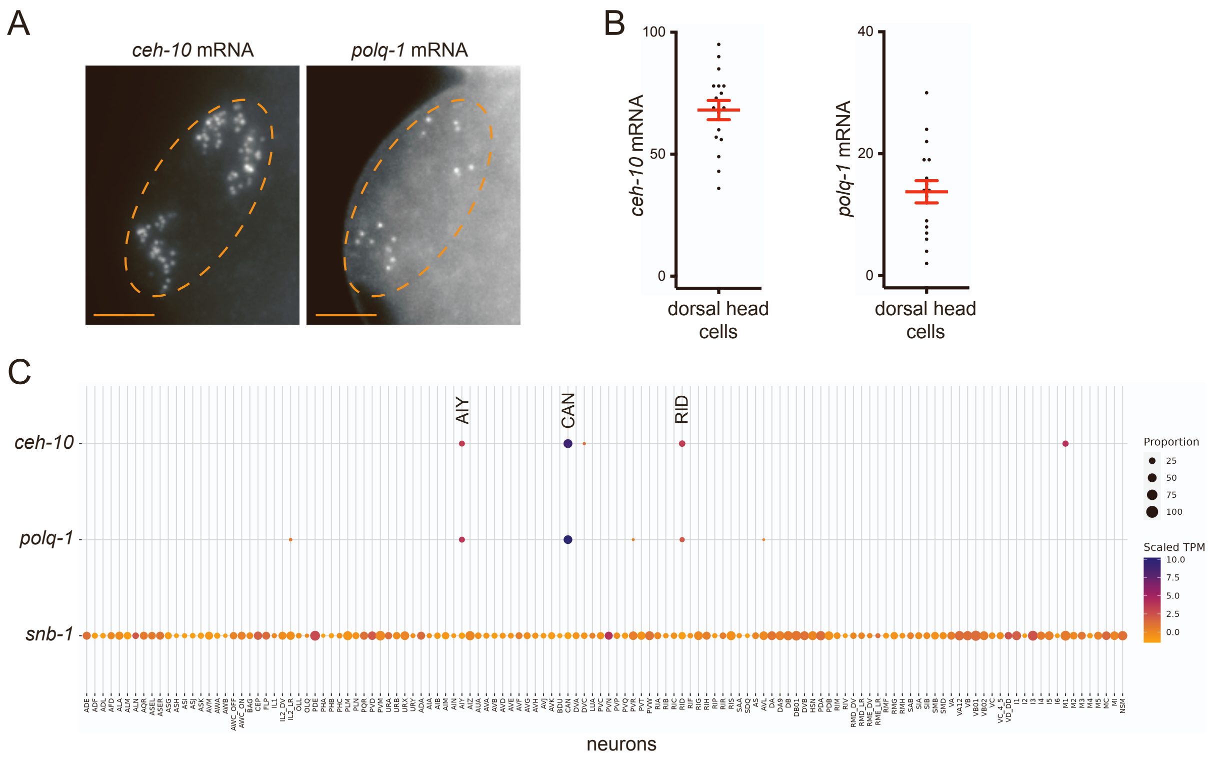

Figure S1. Coexpression of *ceh-10* and *polq-1* at embryonic and larval stages, related to Figure 1.

(A) Detection of *ceh-10* and *polq-1* mRNA by smFISH with exonic probes (labeled with quasar 670 and 570 respectively) in dorsal head cells of 1.5-fold stage embryo; scale bar = 5μm. The same embryo is presented in *ceh-10* and *polq-1* channels.

(B) Quantification of *ceh-10* and *polq-1* mRNA numbers at 1.5-fold stage in the group of *ceh*-10 positive dorsal head cells. Each dot represents quantification in an embryo (n = 17 embryos analyzed in each case). The red bars represent the mean and SEM.

(C) Heatmap of gene expression per neuron type at L4 larval stage generated with the CeNGENApp. For each gene, dot color shows relative expression level across neuron types and dot size shows percent of single cells of each neuron type where expression is detected. *snb-1* is a broadly expressed neuronal gene included for comparison.

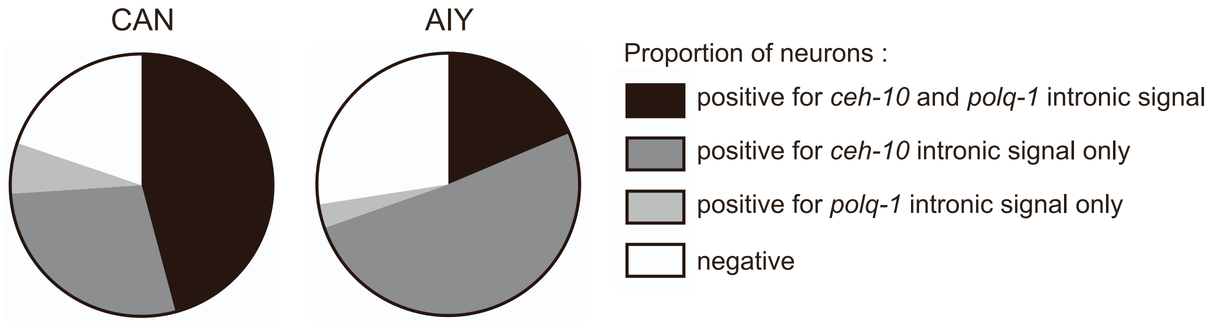

Figure S2. Proportion of nuclei positive for *ceh-10* or *polq-1* nascent RNA, related to Figure 2. Proportion of nuclei of CAN or AIY neurons positive for *ceh-10* or *polq-1* nascent RNA detected by smFISH with intronic probes at 1.5-fold embryonic stage (n = 96 nuclei for CAN, 102 for AIY). Analysis was performed without considering whether intronic signal is from the same locus or not.

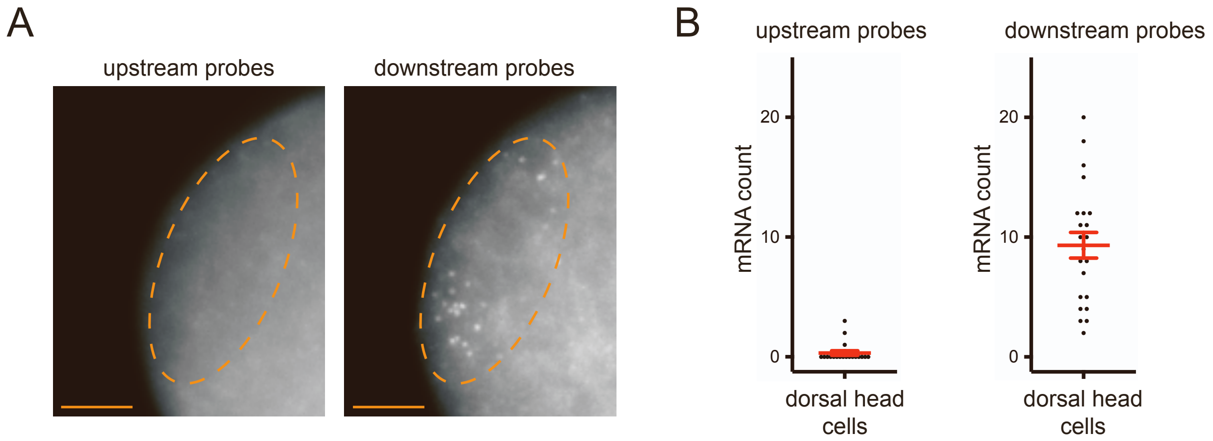

Figure S3. Transcription of *polq-1* in dorsal head cells starts internally, related to Figure 2.
(A) Detection of *polq-1* mRNA using exonic probes located upstream or downstream of *ceh-10* at 1.5-fold embryonic stage in the group of *ceh-10* positive dorsal head cells; scale bar = 5μm.
(B) Quantification of mRNA numbers in the group of *ceh-10* positive dorsal head cells (n = 19 embryos analyzed for upstream, 22 for downstream). The red bars represent the mean and SEM.

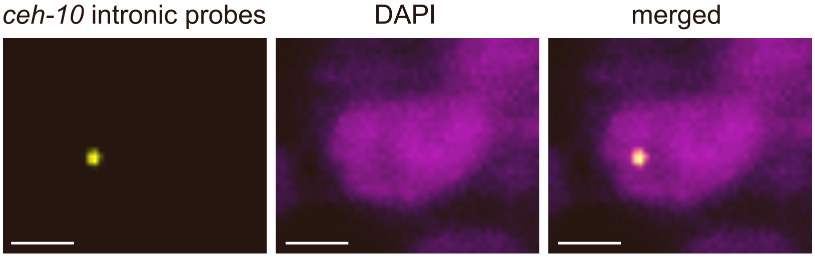

Figure S4. Ectopic expression of TTX-3 and CEH-10 proteins induces endogenous *ceh-10* expression, related to Figure 3.
Detection of endogenous *ceh-10* nascent RNAs by smFISH at the endogenous *ceh-10* transcription site with intronic probes in an *otIs97[unc-119p::ttx-3]; otIs130[unc-119p::ceh-10]* transgenic larva, showing ectopic activation in a non-neuronal nucleus located in the region between the pharynx and the gonad; scale bar = 2μm; n = 5 larvae analyzed.

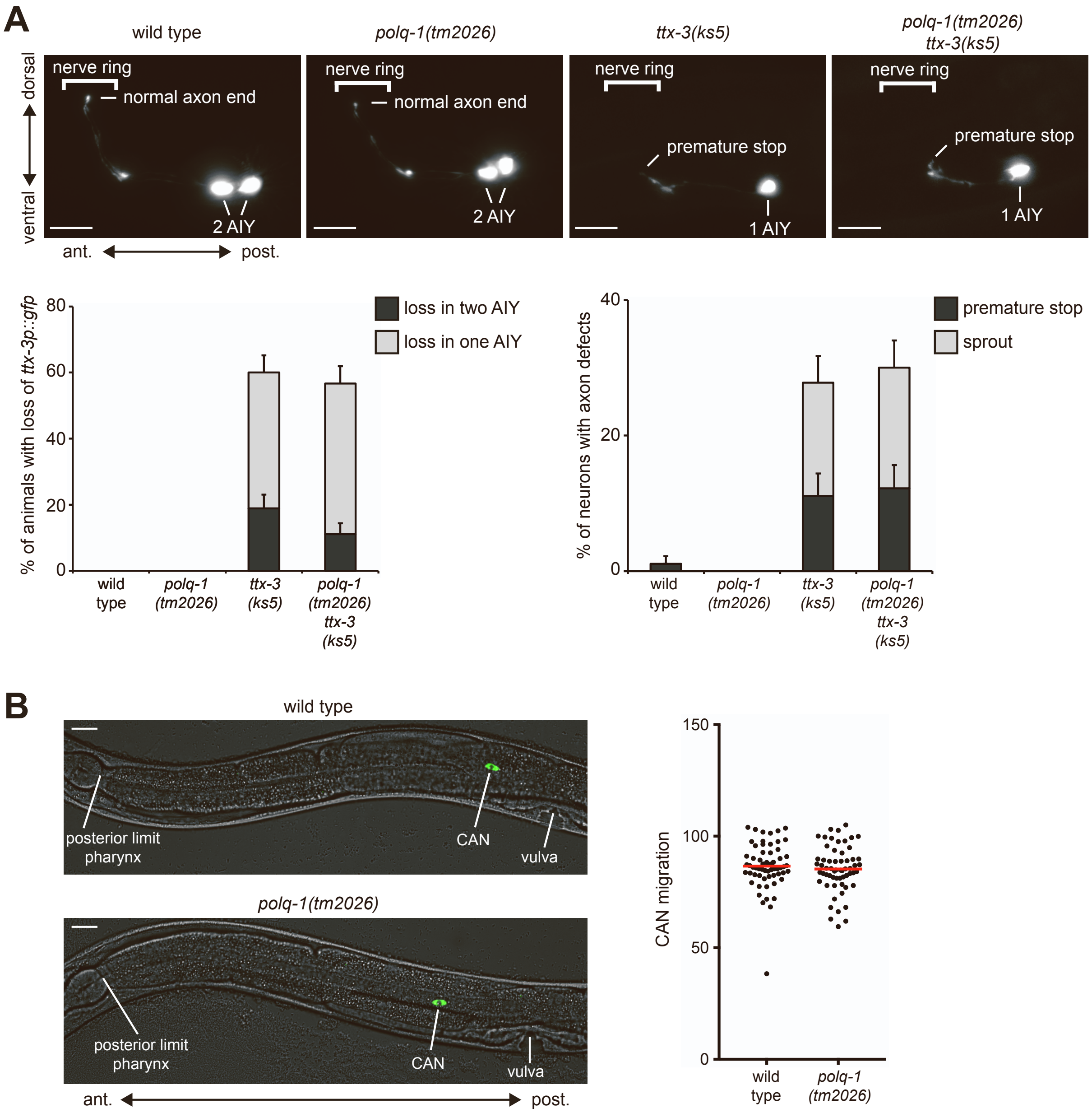

Figure S5. Effect of *polq-1(tm2026)* mutant on AIY and CAN neurons, related to Figure 4.

(A) Effect of *polq-1(tm2026)* mutant on AIY. Pictures: AIY neurons labelled with *ttx-3p::gfp (mgIs18)* in L4 larvae. In wild type and *polq-1(tm2026)* mutant two AIY are detected and have normal axons. In *ttx-3(ks5)* mutant and *polq-1(tm2026); ttx-3(ks5)* double mutant only one AIY is detected and its axon stops prematurely. Lateral view, anterior is left, dorsal is up, scale bar: 10 μm. Left graph: percentage of animals with a loss of *ttx-3p::gfp (mgIs18)* expression in one or both AIY at L4 larval stage. Data are represented as proportion and SEP; n = 90 animals analyzed for each genotype. Right graph: percentage of AIY neurons expressing *ttx-3p::gfp (mgIs18)* with axonal projection defects (premature stop or sprouts) at L4 larval stage. Data are represented as proportion and SEP; n = 90 neurons analyzed for each genotype.

(B) Effect of *polq-1(tm2026)* mutant on CAN. Pictures: CAN neurons labelled with *ceh-23p::gfp (kyIs5)* in L4 larvae. Lateral view, anterior is left, dorsal is up, scale bar: 20 μm. Graph: position of CAN in L4 larvae measured as the distance between the posterior limit of the pharynx and CAN (relative to the distance between the posterior limit of the pharynx and the vulva set to 100). Each dot represents the position of a single CAN neuron, red bars represent the mean, n = 60 neurons analyzed for each genotype.

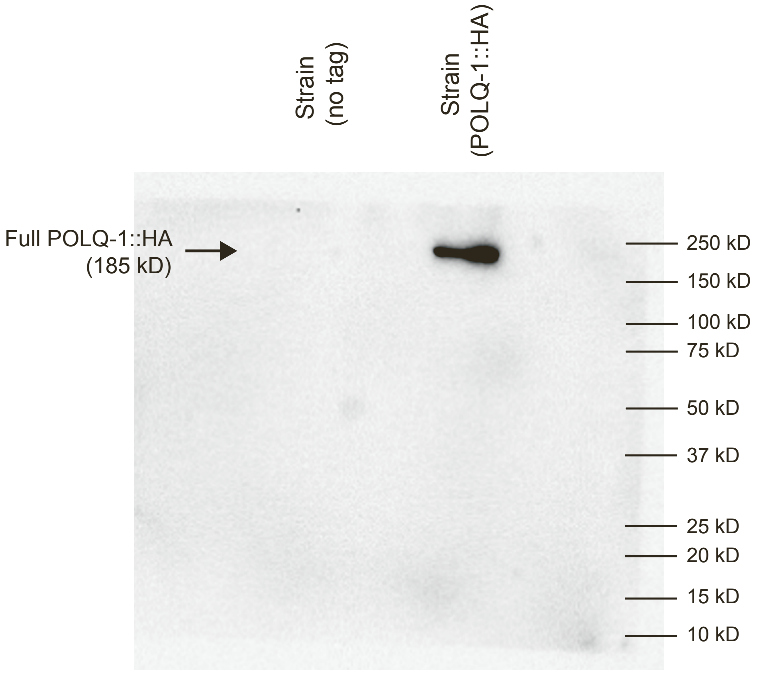

Figure S6. Analysis of POLQ-1::HA by western blot, related to Figure 4.
Anti-HA western blot analysis of embryos of POLQ-1::HA strain (*vba33*) or not tagged strain (N2) (n = two independent repeats).

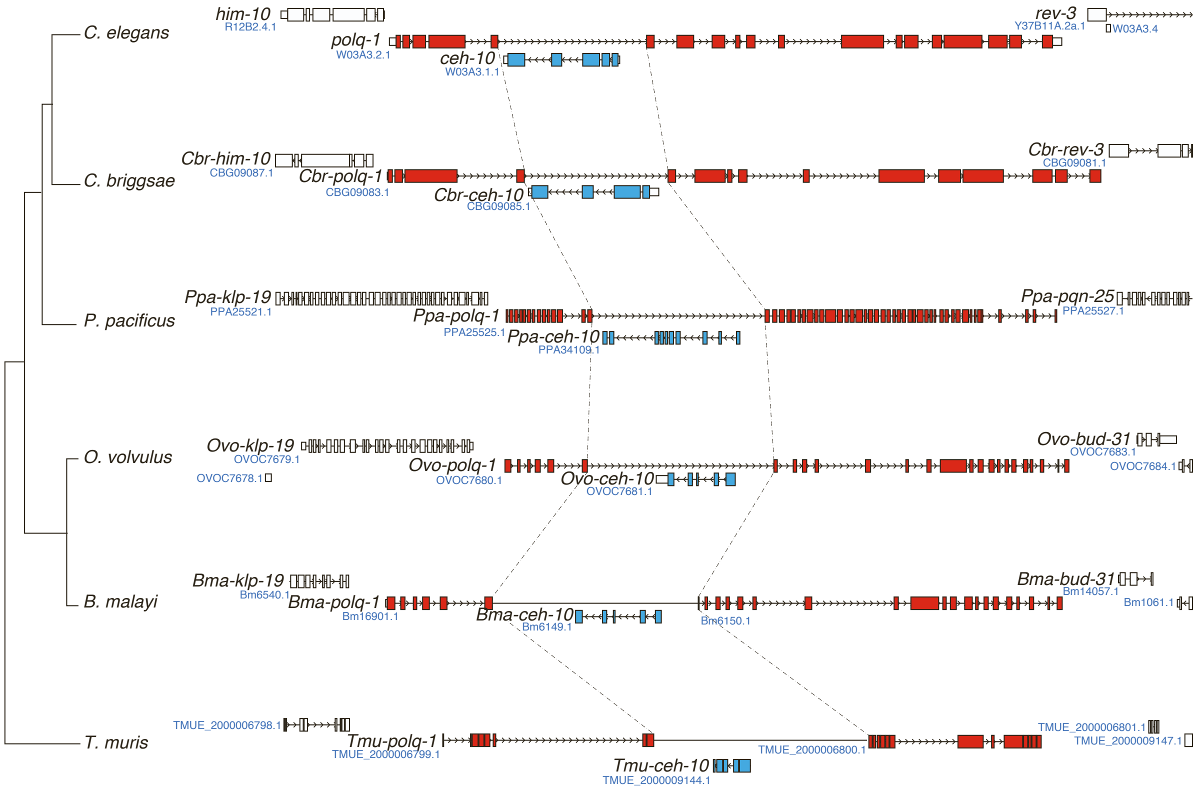

Figure S7. Conservation of *ceh-10* and *polq-1* nested arrangement across nematodes, related to Figure 4.

Across all surveyed nematode species, representing a wide swath of nematode phylogeny (*C. elegans, C. briggsae, P. pacificus* in clade V; *O. volvulus* and *B. malayi* in clade III; *T. muris* in clade I) *ceh-10* and its orthologs (blue) are always nested in reverse orientation within an intron of *polq-1* and its orthologs (red). The dashed line represents conserved exon-intron boundaries flanking *ceh-10*.

Table S2. Sequences of the CRISPR alleles generated, related to STAR methods.

Name	Sequence
polq-1(vba29) deleted sequence (120 bp) in *polq-1* promoter	tcggccgtgaaaactaggccaccgcggccacaaacaaattta gttttcttcgctgaaaaaaacatgtttttcagtctgaaatcagagttttt agtatgaaacaagatggacgaggcttc
polq-1(vba29) sequence targeted by the guide RNA used (PAM underlined)	agtatgaaacaagatggacgagg
ceh-10(vba32) deleted sequence (54 bp) in *ceh-10* enhancer	gcctaatcaacacaaaactgggcagtgtcttctcaaattggctca atttgtgat
ceh-10(vba32) sequences targeted by the two guide RNAs used (PAM underlined)	gggcagtgtcttctcaaattgg<br>tgcctaatcaacacaaaactgg
polq-1(vba33) inserted sequence (linker-3xHA) at C-term of POLQ-1	tctggaggaggaggatcttacccatacgacgttccagactatgcc ggctacccctatgatgtcccggactatgcaggatcttatccatatg acgtcccagattacgct
polq-1(vba33) sequence targeted by the guide RNA used (PAM underlined)	gctgatttaaagtgatatactgg

Table S3. Sequences of the smFISH probes, related to STAR methods.

ceh-10 **exonic**	***polq-1*** **exonic**	***ceh-10*** **intronic**	***polq-1*** **intronic**	***polq-1*** **upstream**	***polq-1*** **downstream**	***gfp***
tttgcattgatgcagcgagc	aatctgccaatcgaacagcg	ctgatttgtgcacttcggaa	cctatttcatcacagcgatt	aaatctacaggaagcctcgt	tcgatcacatttcttgatga	aagttcttctcctttactca
gaattgctcatttgattgct	tcttcaaattgtcttgcctc	atgaactatgaatatcctac	aagggaagagcgggattagc	gctctgcgtaataatcgatg	cgatgattttcagcacggca	gaattgggacaactccagtg
aattgcgaagctcattcgtt	gagccacggagatataagga	ttggtaaggggctacaatga	tcttcagctaaaaagggcca	tcgaacagcgctttgatgtt	acaccttgatcaatcggagt	cccattaacatcaccatcta
cctgaatgcctagaatctca	tctccaacaccgttgaattt	aaaaggggctccgttttcgt	tttgcgggaaaagaagacgt	attcaacacatcaatctgcc	agcatctcgtttccttgtta	cctctccactgacagaaaat
atccgtgagatagatatgca	accaatgaagccacagacag	aaaaagatggatgccgaccg	aatcgccgtttgtcaagtac	gatcttcaaattgtcttgcc	atctgcttaaattagtccgt	gtaagttttccgtatgttgc
gagaaatgttccctgaggac	ccatgtgcatttcatcaacg	aatggaggaaattagcacca	cggtcaggaaacaaccactt	ttgttggcgcactgaatatc	gtgcagattccctctaatat	gtagttttccagtagtgcaa
ccatgaaattgggttgattt	actttttccagtgcagattg	ctctaactttccgagagtca	cagataagcagccacttttc	ttcagctacaatgcttttcc	ctgagatcttgttgtcgtga	acaagtgttggccatggaac
gatactccacaggatccaat	ttcgaaaactttcgcaccgt	tattcttgtgggggagcatg	ccactttgggtaatcttctt	ttgaagcgactttccatgat	ggagaagcttgcagagatca	ggtatctcgagaagcattga
acacagctgtctgtcgaacc	agatttcctcaactccgaac	tcaattgacacattgctgcc	tagtgtgctctcgactactg	acgaagagtacctttctacc	tggcaatgtcagaaagttga	tcatgccgtttcatatgatc
catttccaatagagtcagta	ctcatggaatctcgacgcaa	gtcttgacttgtcttgaagg	aaattggatcatttgcctcc	gagccacggagatataagga	gaattccgaagaagcatctc	gggcatggcactcttgaaaa
cgatacatcagtggagcagg	taacattccgatccttgcaa	aatactatcagccaattggc	gtacggtagacgtcgatctg	accgttgaatttgatgaagt	gttttcgtcttgggagatga	ttctttcctgtacataacct
ctctgatgttgagattggca	atcgtcaatccagcatgatg	accctttagaattttgggtt	ttagtaaccagaagctcctc	gaaatgtcatctcgtctcca	cgagttgtgtgggacttaat	gttcccgtcatctttgaaaa
tctggggctccgaagttata	gtctcgatgattttcagcac	gccaatagaagcgaagaagc	cacttcaaatactccttcct	tggaccaatgaagccacaga	gatgaagcaattgcagctct	tgacttcagcacgtgtcttg
agatgcttgatgagctgaca	tccttgttagaacaccttga	attaggtctcaggtttcagt	agcgggtttttcttcaatc	actcatttggatttgaggcc	aagtgatgcttcaggtggta	taacaagggtatcaccttca
accaccagaagaatttgatg	ctgagatcttgttgtcgtga	agcttttacacaatttgtca	ttgagccgtgaatttcttca	atggtacaaactgccccaag	ctcgactcgccgaattcaaa	ataccttttaactcgattct
ttgtgcgatgtcttctcttc	atctcgatggcaatgtcaga	tgcaaagtcacatcttcaca	cgttctgaaaacttgtagca	ttgtcaaacttgctgctttt	atgaagctcagtatccagtg	gtgtccaagaatgtttccat
atctggtattgagtgaaaat	ctcagtatccagtgcaattg	cctcttcctcataaacaagc	aggccgacatttaattggtt	caatcttctgataatgctcg	gccctcgaattcttgtaaaa	gtgagttatagttgtattcc
cttgaaaagccttctcgagt	tttcactgactccaaccaac	ctctttttctgactcattct	gccttcatttctcggaaaaa	tatcataccaatttcctcaa	gggttacatgccaaaaatgt	gtctgccatgatgtatacat
ggcatagatgtctgggtaat	gctctgatctaacaccgaac	gtccaatttcatgatctttt	cacctcgatcaaaccatctg	ccatgtgcatttcatcaacg	tgccaaacacttacgttgat	atggtctgctagttgaacgc
aattctgtctttccagctag	gtccatcaattccttcaatc	agactaattttgttctacca	taggtccgtattgacctaca	gcaccacgagacgaatcgaa	aaggtgatgccaatcacatt	cgccaattggagtattttgt
atacctgaatccgatcttct	ttctttcaatgttctagccg	gttgttatccggaattatcc	agtccagataaatgtaggca	cgagagcatgtgctcaatgt	gcgattcgtttatgatctga	gtctggtaaaaggacagggc
caggttttctcggtttttcg	tcctcaaatttcggcaactc	taccatttttgtttgttcc	accaattccatatagctctc	attggttccagagcaaaact	tttcactgactccaaccaac	aagggcagattgtgtggaca
attctgccattatggtactt	ttgtcatttcttccatcttc	ccctgaagtttggtttgtaa	cttggttactttttgttggg	attctaactttttccagtgc	cttcgtccttgaagttgatc	tcttttcgttgggatctttc
cgtaccattgctccatatag	gccttcttaggaatacttca	tttgttaggagttttgcacg	attttgttttagctccgagg	aatagttgcactcattccga	gccaacgcagaaaagaatcg	tcaagaaggaccatgtggtc
ttccggaagtggaagagaat	agaatactgcgacttcttcc	tatcatggtgcaccttcaaa	tctcatgtagttttgtcgga	tgcctattcgataaagttct	gctcatttcgctgatcaaat	aatcccagcagctgttacaa
cctcagctgactttgtaatt	aatatggagttggcgattcc	gtcgtaatttctccaatttt	gctctctttctctacaatac	aaaactttcgcaccgtccag	acttgtgagatacctcataa	tatagttcatccatgccatg
agcactttgttgtggatccg	gtaaggatccaggtattgga	ggtgcaatattgtggtgtca	cgcaacacacacagccaagaag	caattggtcgaaatcttgct	agacatccacgaggaattcg	gctcagttggaattctacga
gcattcctaacaaccacgga	ttgcagattttgttggtgga	caggtggttcagggtaatta	ggctcaatttgtgatcatcg	cctcaactccgaaccaatta	gctctgtgattggagtgttt	caagttggtaatggtagcga
gcagcttccatacttttctt	ggatgctttatgactggact	atgggaatgaaatgggtcag	aaactgggcagtgtcttctc	gaactttgttatctccagat	cacaatcattgccgcgtaag	ccctattatttttgacacca
tcactatccgacatatcaca	ccaagcattatgatcatggc		ctcgttttgcctaatcaaca	ggatcttcagagaattctct	catcccagtcgaaggcaaaa	aacttactctgatgacagcg
agttaccggacggtcatcat	tgttcagagggatgatgcat		ccaaatagacgagagcatga	actctcttcggtaagcaata	aagcagtgccttgagatatg	ctcgttagttagtagaactc
ttcacttgacgttgcactgg	ctacagtaaccgcaagtgtt		ttcaacgaggtcagttagtc	aagtctgagaattgcggcga	acaatctcgttgcaaatcca	
cttgttgttgaacatgctca	ggctcgaagagcaagttttt		cctcctcctactcaattttc	cggcatcaagtttcgaagaa	gagagctctgatctaacacc	
aatgatgactgatgttgggg	ttttctcgaagatcggcttc		cattgtctcatttttcgcg	gcaatattcaaagcagtctt	gtccatcaattccttcaatc	
gtgaggaactgacaacaagt	ccgtaagcaagtctccaaat		tgccagatactttgtgtttc	attgatctcatggaatctcg	acgttcatgtaggattcggg	
tgaagcattattgttcggaa	taaattaggccaaggcggta		atttctgtaacttcggtcct	tttccaacaatgagctgtcg	aatgtgacagacacgtcacg	
gagttacatgggattattgt	gtttacatccggaagtttct		ctgtagccgactagcataaa	taatagtccattggctcgtt	ttcgaagaatcgcaagcgga	
	atgaacttggcaagtggcat		ccagataggttgcatttgaa	gttccaatccatgctttata	gagtatggaacagccaatgt	
	aaatccttccagtcgatgta		gtggcagaatgtgagaatta	taacattccgatccttgcaa		
	agacattccatcggacgata		agaaatgggatgaggcaacg	atcgtcaatccagcatgatg		
	cagcatctcgtgggaaattc		tactcattggggataatagg	ttcaatgcattctctttcct		
	cttctatgctcattcttgtc		tgacaccaatgaaggaagga	tatttttctcccgaaatcct		
	actttttcctttgtttcgtt		atgagaatccatgaacaggt	aagtacttgtagccacaaga		
	gtgcgccgaattctttaatt		gtcaactgaactgtcgtgac	agggagatttacgcctgaag		
	cacgtggatttctggacatt		gacagtgtggcgtcataatt	tgagccttgatgagtactcg		
	ttaaatcagcccatgatcgt		ctcctaatagtccaacaaga	agaagtgagagccgatgggc		
			attttcagcggggattttga	ccaccatctgacggtaattt		
			atcccatcaaagggtgtatg	tgtagcatgtcctgttcttc		
